# Public crack cocaine smoking and willingness to use a supervised inhalation facility: implications for street disorder

**DOI:** 10.1186/1747-597X-6-4

**Published:** 2011-02-23

**Authors:** Kora DeBeck, Jane Buxton, Thomas Kerr, Jiezhi Qi, Julio Montaner, Evan Wood

**Affiliations:** 1 British Columbia Centre for Excellence in HIV/AIDS, Vancouver, Canada; 2 School of Population and Public Health, University of British Columbia, Vancouver, Canada; 3 Division of AIDS, Department of Medicine, University of British Columbia, Vancouver, Canada

## Abstract

**Background:**

The health risks of crack cocaine smoking in public settings have not been well described. We sought to identify factors associated with public crack smoking, and assess the potential for a supervised inhalation facility to reduce engagement in this behavior, in a setting planning to evaluate a medically supervised crack cocaine smoking facility.

**Methods:**

Data for this study were derived from a Canadian prospective cohort of injection drug users. Using multivariate logistic regression we identified factors associated with smoking crack cocaine in public areas. Among public crack smokers we then identified factors associated with willingness to use a supervised inhalation facility.

**Results:**

Among our sample of 623 people who reported crack smoking, 61% reported recently using in public locations. In multivariate analysis, factors independently associated with public crack smoking included: daily crack cocaine smoking; daily heroin injection; having encounters with police; and engaging in drug dealing. In sub analysis, 71% of public crack smokers reported willingness to use a supervised inhalation facility. Factors independently associated with willingness include: female gender, engaging in risky pipe sharing; and having encounters with police.

**Conclusion:**

We found a high prevalence of public crack smoking locally, and this behavior was independently associated with encounters with police. However, a majority of public crack smokers reported being willing to use a supervised inhalation facility, and individuals who had recent encounters with police were more likely to report willingness. These findings suggest that supervised inhalation facilities offer potential to reduce street-disorder and reduce encounters with police.

## Background

The use of illicit drugs in public settings, including street, alleys and parks is both a public health and public order concern in many urban areas [1-3]. To date, the use of injection drugs in public settings has received the most attention from policy-makers and public health researchers [2,4,5]. Public injecting is known to present problems for citizens who reside in or around areas where public drug use is prevalent, and scientific studies have documented that using injection drugs in public settings can discourage safer injecting practices resulting in many public health problems, including increased risk for drug overdose events and HIV and other blood-borne infections [6-8]. As a result, some cities have implemented supervised injection facilities which aim to provide an alternative injecting environment that reduces both the health risks associated with injection drug use and the street disorder it can generate [9-13]. While supervised injection facilities have been noted to have measurable success in achieving these public health and public order objectives, the use of inhalable drugs, particularly crack cocaine smoking, has been growing in popularity in many street-based drug scenes [14-16].

In Vancouver, Canada the popularity of crack cocaine and ease of administration through smoking has made public crack cocaine use a common feature of the streets in the city's drug use epicentre, known as the Downtown Eastside [17]. Public crack cocaine smoking is posing a growing burden for law enforcement agencies responsible for maintaining public order [18]. In addition, the health and social harms associated with crack cocaine smoking are extensive. Compared to other drug user populations, crack users are more likely to engage in risky behaviors [19-21] and illegal activities [22,23] and to experience homelessness [14] and health problems [14,24-27], yet are less likely to access health and social services [28]. It has also been recently documented that daily crack cocaine smokers are at a four-fold greater risk of contracting HIV compared to their drug using peers who smoke crack cocaine less often or not at all [16].

Given the dramatic rise in crack cocaine smoking and the public order and public health concerns associated with it, the need for targeted interventions for people who smoke crack cocaine is unambiguous. One potential intervention that is receiving increasing attention from public health officials, health researchers and local community groups is supervised drug consumption facilities analogous to supervised injection sites but that accommodate crack smoking and distribute drug consumption materials specific to safer inhalation (such as sterile crack pipes, mouthpieces, and screens) [16,29-33]. The Canadian Institutes of Health Research recently approved funding to conduct a randomized control trial to evaluate the impact of a supervised inhalation facility on access to medical and social services, particularly addiction treatment, among Vancouver-based crack cocaine smokers [34].

Previous studies have assessed general willingness among local drug users to use a supervised inhalation facility [35,36]; however, these studies were not primarily concerned with street disorder and therefore did not consider the specific risks associated with smoking crack cocaine in public areas nor did they assess willingness to use an inhalation facility among public crack cocaine smokers exclusively. Therefore we conducted a study focused on public crack smokers to identify factors associated with this practice. We also sought to assess willingness to use an inhalation facility among individuals who smoke crack cocaine in public areas to determine the potential impact a supervised inhalation facility might have on street disorder in Vancouver, Canada.

## Methods

Data for this study was obtained from the Vancouver Injection Drug Users Study, which is an open prospective cohort that began enrolling people who inject drugs (IDU) through street outreach as self-referral in May 1996. This study has been described in detail previously [37,38]. In brief, to be eligible participants at recruitment must reside in the Greater Vancouver Regional District, have injected illicit drugs in the previous month, and provide written informed consent. At enrollment and on bi-annual basis participants complete an interviewer-administered questionnaire, and after an examination with a study nurse provide a blood sample for serologic testing. At each study visit participants are provided with a stipend ($20 CDN) for their time. The study has received ethics approval from St. Paul's Hospital and the University of British Columbia's Research Ethics Board. The present analyses are restricted to those participants who reported smoking crack cocaine in the last six months, and were seen for study follow-up during the period of November 2008 and June 2009 as measures for one of our outcomes of interest are available only for this sample period.

In our first analysis among crack cocaine smokers, the outcome of interest was using drugs (non-injection) in public areas in the last six months. As in previous analyses, public areas included city streets, parks, public washrooms, parking lots, clubs or bars and abandoned buildings. To characterize our outcome of interest we *a priori *selected a range of socio-demographic and behavioural variables we hypothesized might be relevant to smoking crack cocaine in public areas. This selection was informed by the 'risk environment framework' and previous analyses among street-involved drug users highlighting connections between social, structural and environmental level factors and risky drug consumption practices [14,31,39-41]. Variables included: age (per year older); gender (female vs. male); Aboriginal Ancestry (yes vs. no); limited access to private space, defined as answering "no" to the question: "Do you have a private indoor space for socializing with friends and acquaintances?" or reporting that the number of guests they were allowed to have in their residence at one time was restricted to less than three (yes vs. no); daily cocaine injection (yes vs. no); daily heroin injection (yes vs. no); daily crack cocaine smoking (yes vs. no); non-fatal overdose, self identified by participants (yes vs. no); encounters with police in the last month, defined as being questioned, searched or stopped by police (yes vs. no); being a victim of violence defined as being physically assaulted (yes vs. no); sex trade involvement, defined as exchanging sex for money, shelter, drugs or other commodities (yes vs. no), and participation in drug dealing (yes vs. no). Unless otherwise stated, all drug use and behavioural variables refer to the previous six month period.

In a second analysis, we sought to assess and identify predictors of willingness to use a supervised inhalation room. Because we were particularly concerned with public drug use we restricted our sample to crack cocaine smokers that reported recently using non-injection drugs in public areas. To measure willingness we asked participants "If there was a safe place to smoke your drugs (ventilated inhalation room), close to where you buy or use, would you use it?"

Variables of interest for our second analysis were also selected *a priori *based on factors we hypothesized might be associated with willingness to use an inhalation room. These included age (per year older); gender (female vs. male); Aboriginal Ancestry (yes vs. no); limited access to private space, as defined above (yes vs. no); drug scene exposure, defined as spending an average of seven or more hours on the street each day in Vancouver's drug use epicentre in the previous six months (yes vs. no); most drug use in public areas, defined based on reports that public locations were where they most frequently used drugs (yes vs. no); daily crack cocaine smoking (yes vs. no); risky pipe sharing, defined as reporting sharing a crack pipe or mouthpiece in the same six month period as having burns or sores on their mouth (yes vs. no); encounters with police in the last month (yes vs. no); and being a victim of violence (yes vs. no). As above, unless otherwise stated, all drug use and behavioural variables refer to the previous six month period.

For both of our first and second analyses, we used univariate and multivariate statistics to determine factors associated with our outcomes of interest. In univariate analysis categorical explanatory variables were analyzed using Pearson's chi-square test and continuous variables were analyzed using the Wilcoxon rank sum test. Fisher's exact test was used when one or more of the cell counts was less than or equal to five. To evaluate factors independently associated with our outcomes of interest, all variables that were *p *< 0.05 in univariate analyses were entered into the respective multivariate regression models. All statistical analyses were performed using SAS software version 9.1 (SAS, Cary, NC). All p-values are two sided.

## Results

During the study period 623 participants were seen for study follow-up visits and reported smoking crack cocaine in the last six months. These included 249 (40%) women and 231 (37%) persons who identified as Aboriginal. The median number of times that participants reported smoking crack cocaine in an average day was 4 (interquartile range = 2-10). Among our sample of 623 crack smokers, a total of 382 (61%) reported using in public areas in the last six months. The characteristics of the study sample stratified by public drug use are presented in Table 1, and the univariate analyses of behavioral and socio-demographic variables associated with public drug use among crack cocaine smokers are presented in Table 2. The results of the multivariate logistic regression for factors associated with public drug use among crack cocaine smokers are also shown in Table 2. Factors that remained independently associated with our outcome of interest included: daily heroin injection, daily crack cocaine smoking, encounters with police and drug dealing (see Table 2).

**Table 1:** Characteristics of crack cocaine smokers stratified by public drug use (n = 623)

	**Public drug use**^**a**^	
	
*Characteristic*	Yes *n*= 382, *n *(%)	No *n*= 241, *n *(%)
**Age pre year older**		
(Median, IQR)^**c**^	43 (37-49)	46 (40-50)
***Female Gender***		
Yes	145 (38)	104 (43)
No	237 (62)	137 (57)
***Aboriginal Ancestry***		
Yes	137 (36)	94 (39)
No	245 (64)	147 (61)
***Limited Access to Private Space***^***d***^		
Yes	316 (83)	169 (70)
No	66 (17)	72 (30)
**Daily Cocaine Injection **^**d**^		
Yes	39 (10)	9 (4)
No	343 (90)	232 (96)
**Daily Heroin Injection **^**d**^		
Yes	107(28)	25 (10)
No	275 (72)	216 (90)
**Daily Crack Smoking **^**d**^		
Yes	220 (58)	72 (30)
No	162 (42)	169 (70)
**Overdose (non-fatal)**^**d**^		
Yes	18 (5)	3 (1)
No	364 (95)	238 (99)
**Encounters with police **^**e**^		
Yes	114 (30)	35 (15)
No	268 (70)	206 (85)
**Victim of Violence **^**d**^		
Yes	77 (20)	30 (12)
No	305 (80)	211 (88)
**Sex Trade **^**d**^		
Yes	60 (16)	19 (8)
No	322 (84)	222 (92)
**Drug Dealing **^**d**^		
Yes	150 (39)	46 (19)
No	232 (61)	195 (81)

Note: ^a ^Public locations include: city streets, parks, public washrooms, parking lots, clubs or bars, and abandon buildings; ^c ^IQR = Inter Quartile Range; ^d ^Denotes activities or situations referring to previous 6 months; ^e ^Denotes activities or situations referring to previous month.

**Table 2:** Univariate and multivariate analyses of factors associated with public drug use among crack cocaine smokers^a ^(n = 623)

	Univariate		Multivariate	
	
*Characteristic*	**OR**^**b **^**(95% CI)**	***p-value***^c^	**AOR (95% CI**)	*p-value*
***Older Age***				
Per year older	**0.96 (0.94 - 0.98)**	**<0.001**	0.98 (0.96 - 1.00)	0.065
***Gender***				
Female vs. Male	0.81 (0.58 - 1.12)	0.197		
***Aboriginal Ancestry***				
Yes vs. No	0.87 (0.63 - 1.22)	0.429		
**Limited Access to Private Space **^**d**^				
Yes vs. No	**2.04 (1.39 - 2.99)**	**<0.001**	1.49 (0.99 - 2.26)	0.058
**Daily Cocaine Injection **^**d**^				
Yes vs. No	**2.93 (1.39 - 6.17)**	**0.003**	1.70 (0.77 - 3.75)	0.190
**Daily Heroin Injection **^**d**^				
Yes vs. No	**3.36 (2.10 - 5.38)**	**<0.001**	**1.95 (1.17 - 3.27)**	**0.011**
**Daily Crack Cocaine Smoking **^**d**^				
Yes vs. No	**3.19 (2.26 - 4.49)**	**<0.001**	**2.17 (1.49 - 3.14)**	**<.001**
Overdose (non-fatal)* ^**d**^				
Yes vs. No	**3.92 (1.13 - 20.98)**	**0.020**	2.04 (0.55 - 7.61)	0.288
**Encounters with Police **^**e**^				
Yes vs. No	**2.50 (1.64 - 3.81)**	**<0.001**	**1.69 (1.07 - 2.68)**	**0.025**
**Victim of Violence **^**d**^				
Yes vs. No	**1.78 (1.12 - 2.80)**	**0.013**	1.52 (0.92 - 2.51)	0.100
**Sex Trade **^**d**^				
Yes vs. No	**2.18 (1.26 - 3.75)**	**0.004**	1.30 (0.72 - 2.38)	0.386
**Drug Dealing **^**d**^				
Yes vs. No	**2.74 (1.87 - 4.01)**	**<0.001**	**1.61 (1.06 - 2.47)**	**0.027**

Note: ^a ^Public areas included: city streets, parks, public washrooms, parking lots, clubs or bars, and abandon buildings; ^b^OR = Odds Ratio, CI = Confidence Interval; AOR = Adjusted Odds Ratio; ^c^Unless otherwise stated, values are based on Pearson's chi-square test for categorical variables and Wilcoxon rank sum test for continuous variables with 1 degree of freedom; ^d ^Denotes activities or situations referring to previous 6 months; ^e ^Denotes activities or situations referring to previous month. *p-value and 95% CI reported from Fisher's Exact Test as 25% of cells had expected counts less than 5.

For our second analysis, the demographic and behavioural characteristics of public crack cocaine smokers stratified by willingness to use a supervised inhalation room are presented in Table 3, and the univariate results of factors associated with willingness to use a supervised inhalation room are presented in Table 4. The results of the multivariate logistic regression for factors associated with willingness to use a supervised inhalation room are also shown in Table 4. Factors that remained independently associated with willingness included: female gender, risky pipe sharing and recent encounters with police (see Table 4.).

**Table 3:** Characteristics of crack cocaine smokers who use drugs in public stratified by willingness to use a supervised inhalation room (n = 382)

	**Willing to use SIR **^a^	
	
*Characteristic*	Yes *n*= 271, *n *(%)	No *n*= 111, *n *(%)
**Age pre year older**		
(Median, IQR)^c^	43 (37-49)	44 (37-48)
***Female Gender***		
Yes	117 (43)	28 (25)
No	154 (57)	83 (75)
***Aboriginal Ancestry***		
Yes	108 (40)	29 (26)
No	163 (60)	82 (74)
**Limited Access to Private Space **^**e**^		
Yes	230 (85)	86 (77)
No	41 (15)	25 (23)
**Drug Scene Exposure **^**e, f**^		
Yes	157 (58)	49 (44)
No	114 (42)	62 (56)
**Most Drug Use in Public Areas **^**e**^		
Yes	140 (52)	43 (39)
No	131 (48)	68 (61)
**Daily Crack Cocaine Smoking **^**e**^		
Yes	164 (61)	56 (50)
No	107 (39)	55 (50)
**Risky Pipe Sharing **^**e**^		
Yes	38 (14)	3 (3)
No	233 (86)	108 (97)
**Encounters with Police **^**d**^		
Yes	92 (34)	22 (20)
No	179 (66)	89 (80)
**Victim of Violence **^**e**^		
Yes	53 (20)	24 (22)
No	218 (80)	87 (78)

Note: ^c ^IQR = Inter Quartile Range; ^d ^Denotes activities or situations referring to previous 6 months; ^e ^Denotes activities or situations referring to previous month; ^f ^Drug scene exposure was defined as spending an average of 7 or more hours on the street each day in Vancouver's drug use epicenter in the previous six months

**Table 4:** Univariate and multivariate analyses of factors associated with willingness to use a supervised inhalation room among participants that smoke crack cocaine and use drugs in public locations (n = 382)

	Univariate		Multivariate	
	
*Characteristic*	**OR**^**a **^**(95% CI)**	***p-value***^**b**^	**AOR**^**c **^**(95% CI**)	*p-value*
***Older Age***				
Per year older	1.01 (0.98 - 1.04)	0.446		
***Gender***				
Female vs. Male	**2.25 (1.38 - 3.68)**	**0.001**	**2.11 (1.26 - 3.55)**	**0.005**
***Aboriginal Ancestry***				
Yes vs. No	**1.87 (1.15 - 3.05)**	**0.011**	1.61 (0.96 - 2.72)	0.072
**Limited Access to Private Space **^**d**^				
Yes vs. No	1.63 (0.94 - 2.84)	0.083		
**Drug Scene Exposure **^**d**^				
Yes vs. No	**1.74 (1.12 - 2.72)**	**0.014**	1.40 (0.86 - 2.28)	0.181
**Most Drug Use in Public Areas **^**d**^				
Yes vs. No	**1.69 (1.08 - 2.65)**	**0.022**	1.39 (0.85 - 2.28)	0.187
**Daily Crack Cocaine Smoking **^**d**^				
Yes vs. No	1.51 (0.96 - 2.35)	0.071		
**Binge Drug Use **^**d**^				
Yes				
Risky Pipe Sharing* ^**d**^				
Yes vs. No	**5.87 (1.79 - 30.29)**	**<0.001**	**5.50 (1.63 - 18.56)**	**0.006**
**Encounters with Police **^**e**^				
Yes vs. No	**2.08 (1.22 - 3.53)**	**0.006**	**2.09 (1.20 - 3.65)**	**0.010**
**Victim of Violence **^**d**^				
Yes vs. No	0.88 (0.51 - 1.52)	0.648		

Note: ^a^OR = Odds Ratio, CI = Confidence Interval; ^b^Unless otherwise stated, values are based on Pearson's chi-square test for categorical variables and Wilcoxon rank sum test for continuous variables with 1 degree of freedom; ^c^AOR = Adjusted Odds Ratio; ^d^Denotes activities or situations referring to previous 6 months; ^e ^Denotes activities or situations referring to previous month. *p-value and 95% CI reported from Fisher's Exact Test as 25% of cells had expected counts less than 5.

## Discussion

We found that the majority of crack cocaine smokers in our study reported having used drugs in public areas at some point in the last six months. This group was more likely to be higher-intensity drug users with respect to heroin injection and crack cocaine smoking, have encounters with the police and be involved in drug dealing. Of these public crack cocaine smokers, 71% reported being willing to use a supervised inhalation room if one was available. Individuals who reported being willing were more likely to be female, engage in risky pipe sharing and have encounters with the police.

The profile of public crack cocaine smokers as higher-intensity drug users who have interactions with the criminal justice system is reflective of previous findings describing public injection drug user populations [1,5]. The association between drug dealing and public crack use may reflect the increased amount of time individuals spend on the street when engaged in street-level drug dealing. It may also be a function of the accessibility of drugs and additional resources gained through drug dealing which may lead to greater drug consumption and hence a greater likelihood for consuming in public areas [23].

Our finding that 71% of public crack cocaine smokers are willing to use an inhalation facility also supports previous willingness estimates conducted among the general population of Vancouver-based illicit drug users and suggests that an intervention of this nature will likely reach the target population [36]. The high degree of willingness that this study found among public crack cocaine smokers to use an inhalation facility suggests that, like supervised injection facilities, these interventions are likely to successfully encourage public drug users to relocate to indoor venues.

The increased likelihood of being willing to use an inhalation facility among female participants may reflect heightened vulnerability of women involved in street drug use and it is noteworthy that Vancouver's supervised injection facility has had success in attracting vulnerable female drug users and providing them with safer alternatives to street-based drug using venues. In previous research female IDU have described the unique role that Vancouver's supervised injection facility has played in promoting their physical security and health safety [42].

Interestingly, one of the common features among both public crack cocaine smokers and those who are willing to use a supervised inhalation facility is their elevated likelihood of recently having encounters with law enforcement. This suggests that public crack cocaine smokers who are the subject of law enforcement attention are very willing to relocate to alternative off-street and health-focussed environments if they were made available. Indeed, our data indicate that 81% of public crack cocaine smokers who have had a recent encounter with police are willing to use a supervised inhalation facility.

A key implication of these findings is that there is a large demand for supervised inhalation rooms among individuals that are potentially key contributors to drug-related street disorder. The association between public crack smoking and encounters with police suggests that interventions of this nature are likely to target a critical sub-population of drug users and could be a valuable tool for police in the management of street disorder. Previous studies have found that Vancouver police regularly refer public injection drug users to the local supervised injection facility [43]. Since our analysis indicates that local police are already frequently interacting with public crack smokers the establishment of a supervised inhalation facility could provide a unique opportunity for police to direct this vulnerable group to a low-threshold service where they will have opportunities to be linked with appropriate health and social services.

It is critical to note that although this study suggests that supervised inhalation facilities could aid in the reduction of public disorder, drug consumption facilities do not address the route causes of street disorder and are not appropriate substitutes for other essential health and social interventions such as supportive housing and addiction treatment. To be effective supervised inhalation facilities should be integrated into broader comprehensive approaches to addressing the problems associated with illicit drug addiction.

This study has a number of limitations. Firstly, VIDUS is a community recruited non-randomized sample and therefore our findings may not be generalizable to other settings. If supervised inhalation facilities are being considered in other settings, willingness studies should be conducted among the local target population and should not rely on the findings emerging from our setting. The generalizability of our findings is also limited by our study sample which was restricted to individuals with a history of injection drug use. Crack cocaine smokers who did not have a history of injection drug use were not eligible for our study. Given the harms associated with injection drug use we anticipate that if a selection effect were present it would likely bias our sample towards high risk drug users, suggesting that this group would be an appropriate target population for public health intervention. We should also note that among our study sample daily crack cocaine smoking was significantly more common than daily injecting, suggesting that despite the requirement of a history of injecting, our sample represents a primarily crack cocaine smoking population. Secondly, some of our measures relied on self-report and could be vulnerable to socially desirable reporting. This would have likely been of most relevant to our measure of willingness, since respondents might perceive a pressure to report being willing to engage with low-threshold services of this nature given the widespread activism among local drug users in our study setting to implement supervised drug consumption facilities [32]. While it is possible that some respondents may over-report willingness, a previous study comparing measures of willingness to use a supervised injection facility before it was established with later reports of actual attendance after an injection facility was established suggests that willingness measures are good predictors of later behaviour among this population [44]. Lastly, socially desirable reporting could have influenced reports of stigmatized behaviour, such as public drug use leading to an underestimation of public crack smoking. If social desirability was an issue in our study we suspect our finding would be a conservative indication of the prevalence of and harms associated with public drug use among crack cocaine smokers.

In summary, our study found that locally public crack smoking is a common practice that is also associated with recent encounters with police. We found that the majority of public crack smokers were willing to use an inhalation facility if one were available. Furthermore, public crack smokers who had recent encounters with police were even more likely to be willing to use an inhalation room, suggesting that supervised inhalation facilities may offer unique opportunities to decrease one component of drug-related street disorder and reduce the burden on local law enforcement agencies.

## Competing interests

**JM **has received grants from, served as an ad hoc advisor to, or spoke at various events sponsored by; Abbott, Argos Therapeutics, Bioject Inc, Boehringer Ingelheim, BMS, Gilead Sciences, GlaxoSmithKline, Hoffmann-La Roche, Janssen-Ortho, Merck Frosst, Pfizer, Schering, Serono Inc, TheraTechnologies, Tibotec, Trimeris.

Authors declare no other competing interests.

## Authors' contributions

The specific contributions of each author are as follows: KD, TK, and EW were responsible for study design; JQ conducted the statistical analyses; KD prepared the first draft of the analysis; TK, JB, JM and EW contributed to the main content and provided critical comments on the final draft. All authors approved the final manuscript.

## Funding

The study was supported by the US National Institutes of Health (R01DA011591) and (R01DA021525) and the Canadian Institutes of Health Research (MOP-79297, RAA-79918). Thomas Kerr is supported by the Michael Smith Foundation for Health Research and the Canadian Institutes of Health Research. Kora DeBeck is supported by a Michael Smith Foundation for Health Research Senior Graduate Trainee Award and a Canadian Institutes of Health Research Doctoral Research Award. Julio Montaner has received an Avant-Garde award (DP1DA026182) from the National Institute of Drug Abuse, US National Institutes of Health.
